# Bioacoustic Monitoring Reveals Patterns of Landscape Use by Migrating Birds at a Great Lakes Barrier Crossing

**DOI:** 10.1002/ece3.72635

**Published:** 2025-12-16

**Authors:** Zach G. Gayk, Benjamin M. Van Doren

**Affiliations:** ^1^ Department of Natural Resources and Environmental Sciences University of Illinois Urbana‐Champaign Urbana Illinois USA

**Keywords:** acoustic monitoring, barrier crossing, bird migration, landscape use, movement ecology

## Abstract

Understanding how highly mobile animals use landscapes at broad geographic scales remains a major challenge in ecology. Traditional monitoring approaches often lack the spatial and temporal resolution to monitor how migratory species use heterogeneous landscapes, contend with movement barriers, and interact with urban and developed landscapes. Here, we use a passive acoustic monitoring network to characterize landscape use of migrating songbirds in the Keweenaw peninsula, a major barrier crossing point along the south shore of Lake Superior. Using nearly 3 million acoustic detections of migrants from 18 sites spanning 328 km^2^, we demonstrate that landscape use is shaped strongly by local geography and wind conditions during reoriented movements associated with barrier crossing. Generally, more songbirds used the peninsula during wind conditions favorable for migration. Following winds unfavorable for crossing in spring, birds concentrated in coastal and ridge landscapes oriented along an east–west axis. Geographic gradients and coastline orientation both played important additional roles in shaping migrants' landscape use. Together, our results illustrate the complex role of large water barriers in shaping landscape use in highly mobile animals. More broadly, our findings demonstrate the value of acoustic monitoring as a novel technique for studying migratory animals' landscape use. This approach offers a powerful, scalable tool that can be deployed across complex landscapes and inform conservation priorities of hard‐to‐monitor species.

## Graphical abstract

We use a broad geographic‐scale passive acoustic monitoring network to characterize landscape use of migrating songbirds in the Keweenaw peninsula, a major barrier crossing point along the south shore of Lake Superior. Using nearly 3 million acoustic detections of migrants from 18 sites spanning 328 km^2^, we demonstrate that landscape use is shaped strongly by local geography and wind conditions, including reoriented movements associated with barrier crossing. Our results illustrate the complex role of large water barriers in shaping broad geographic scale patterns of landscape use of highly mobile animals. More broadly, our findings demonstrate the value of acoustic monitoring as a novel technique for geographic scale analyses of migratory animals' landscape use.

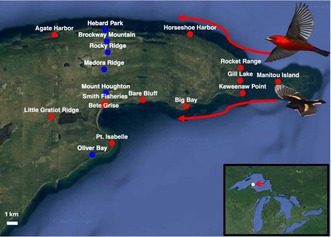

## 1 Introduction

How animal populations utilize space and geographic features of the landscape is a fundamental question in ecology, with broad implications for biogeography, population ecology, behavior, and conservation (Kernohan et al. 2001). Although the spatial landscape use of animals is well studied for many large terrestrial animals and commercial fish populations (Meyer and Holland 2005; Moorcroft 2012), the landscape use of highly mobile and migratory organisms is relatively poorly known (Afonso et al. 2009). This knowledge gap hinders ecological study, conservation, and management.

Birds are an ideal system for studying landscape use in mobile organisms. Approximately one in five of the world's bird species is migratory, and many traverse thousands of kilometers during migration (Somveille et al. 2013). Migration is fraught with risk and has significant fitness consequences (Sillett and Holmes 2002). During migration, birds must navigate across spatially heterogeneous landscapes offering varying risks and benefits (Schmaljohann et al. 2022). Locating high‐quality stopover habitat with abundant resources is critical for migratory birds to gain sufficient fat reserves necessary to complete dangerous migrations (Smolinsky et al. 2013; Gómez et al. 2019). Amidst precipitous population declines in migratory birds in the past 50 years (Rosenberg et al. 2019), it is critical to characterize how these species utilize landscapes during migration periods and prioritize regions of importance for stopover and passage. However, it has proven extremely challenging to measure bird movements across landscapes at sufficiently broad scales and sufficiently fine resolution to inform conservation action, especially for the small, highly mobile songbirds that represent the majority of migratory species.

During migration, birds must often contend with dangerous geographic barriers such as oceans, lakes, and deserts (Schmaljohann et al. 2009; Adamík et al. 2016; Aschwanden et al. 2020). Landscapes immediately preceding and following barrier crossings are important locations for individuals to acquire sufficient fuel to cross, recover, and refuel after reaching the other side (Moore and Kerlinger 1987; Smolinsky et al. 2013; Lavallée et al. 2021). The Great Lakes of North America are an important barrier for migratory birds and shape the migration trajectories of billions of individuals (Archibald et al. 2017). Consequently, the coastlines of the Great Lakes are among the most heavily used landscapes in North America by migratory birds (Ewert et al. 2011; Cohen et al. 2022). These lands simultaneously experience significant pressure from urban development and include numerous major cities. These competing needs have led one such area, Chicago, to be identified as the single United States city with the highest risk for urban impacts on migratory birds from artificial light (Horton et al. 2019).

Passive acoustic monitoring is a rapidly evolving method for monitoring wildlife populations, and this approach is increasingly being adopted to study migratory animals (Ross et al. 2023; Van Doren et al. 2023). The adoption of machine learning methods is transforming acoustic monitoring and its use as a method for understanding migration (Van Doren et al. 2024), facilitating low‐cost monitoring at finer spatial and taxonomic resolution than can currently be offered by other techniques, such as radar. However, no studies have yet applied acoustic monitoring to investigate how migratory birds use spatially heterogeneous landscapes during migration. Such an approach could reveal how migrant populations vary in their habitat use across geographies, how weather conditions influence landscape use, and which regions of high‐intensity usage should be given conservation priority.

Here, we used acoustic monitoring to investigate the aerial and terrestrial landscape use of migratory songbirds (order Passeriformes) in a region of high migration traffic along the coast of Lake Superior, the largest freshwater lake in the world. The Keweenaw peninsula of Michigan, USA is the longest peninsula extending from the south shore of Lake Superior and is a well‐known concentrator of migratory birds attempting to cross the lake (Wood 1931; Binford 2006; Kaplan and Youngman 2011). However, how migratory birds use the peninsula's varied landscapes, especially before and after water crossings under different environmental conditions, is currently unknown. Such information is important in the context of expanding commercial forestry and recreational tourism industries, which present unquantified threats to migratory bird passage in the region.

In this study, we addressed two primary objectives. First, we identified the regions of the Keweenaw peninsula of greatest importance for songbird migrants contending with a large migration barrier. Second, we examined how atmospheric conditions and geography interact to shape landscape usage. For objective one, we predicted that songbird migrants would concentrate in areas closest to the Lake Superior shoreline, especially the areas closest to the tip of the peninsula, where they are likely to first make landfall when conditions are not conducive for crossing the lake. We also expected higher concentrations in areas with coastlines oriented along an east–west axis, as this orientation facilitates migrants' movement toward the mainland. For objective two, we expected that migratory birds would use the study area more heavily following offshore westerly winds (i.e., winds blowing from the west) or headwinds, which may drift migrants off of their intended north–south courses (Archibald et al. 2017; Gagnon et al. 2022), impede barrier crossing of Lake Superior, and result in higher use of adjacent coastal areas.

**FIGURE 1:**
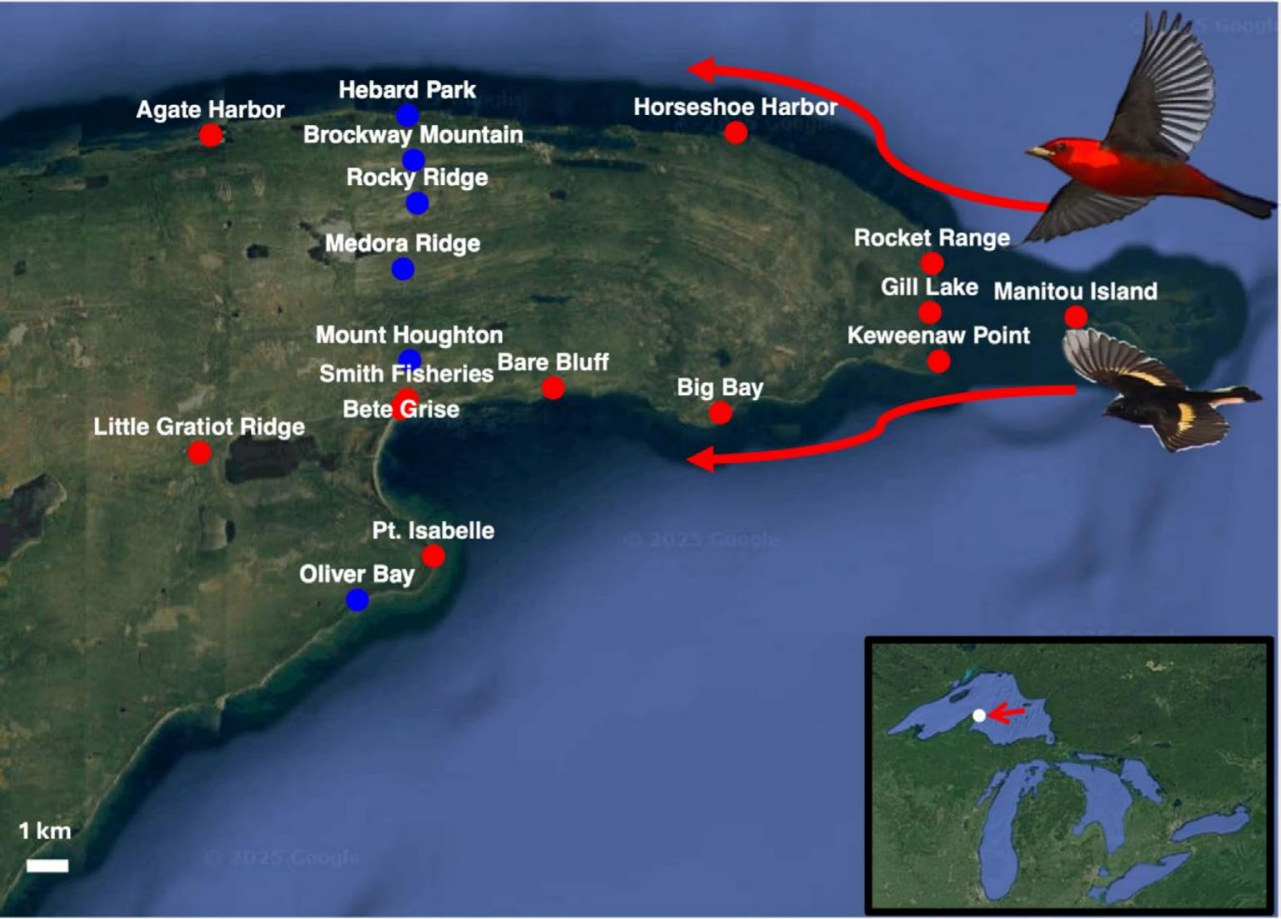
Locations of 18 acoustic recordings sites in the Keweenaw peninsula, Michigan, USA where reoriented songbird migration and spatial landscape use patterns were studied. Red sites indicate sites studied in spring 2022–fall 2024, while blue sites indicate sites studied only in 2024. Red arrows indicate migrants' westerly flight directions away from Lake Superior. Images of Scarlet Tanager ( *Piranga olivacea* ) and American Redstart ( *Setophaga ruticilla* ) used with permission of photographer, Skye Haas. Map produced via ggmap R package, with permission from Google. White bar indicates scale of 1 km.

We expected predominantly westward movements of songbirds in our study region, as it has been documented that migrants move away from Lake Superior post‐dawn in a westward direction in both spring and fall (Binford 2006; Kaplan and Youngman 2011; Gayk and Mennill 2020). However, the exact cause of this unidirectional movement phenomenon, the variation in geographic patterns of movement across the peninsula, and the relationship between movement and weather have not been studied (Figure 1). We also expected that wind conditions would produce usage intensity gradients across the study area. For example, westerly winds might result in heavier use of western study sites if migrants correct for wind drift by reorienting westward, into the wind. We investigated these questions for a suite of migrant songbirds in order Passeriformes with a focus on three individual families: wood‐warblers (Parulidae), sparrows (Passerellidae), and thrushes (Turdidae). Our results demonstrate that passive acoustic monitoring is a viable tool for studying broad landscape use of migratory birds and show how environmental conditions drive patterns of migrant landscape use in a dynamic barrier system.

## 2 Methods

### 2.1 Study Site and Migration Behavior

The Keweenaw peninsula is a 120‐km peninsula that extends northeast from the south shore of Lake Superior (Figure 1). Nocturnally migrating songbirds have been anecdotally observed to engage in diurnal migration (termed “morning flights”) in this region, wherein mixed species groups of songbirds move westward along the Lake Superior shoreline, irrespective of the season, after successful or failed crossings of Lake Superior (Binford 2006). Traditionally, acoustic monitoring of bird migration takes place during nocturnal migration (Sanders and Mennill 2014; Béasse et al. 2025), but we chose to monitor diurnal morning flights for several reasons. During nocturnal migration, birds are distributed broadly over landscapes (Lavallée et al. 2021) and cross water bodies such as Lake Superior. However, birds in morning flight tend to be concentrated in defined regions of the landscape as they reorient to land and search for foraging or resting habitat (Wiedner et al. 1992). Observations from a single study site suggest that morning flight migrants in the Keweenaw peninsula fly along the shoreline, primarily within 300 m of the coast, and use a wide array of the peninsula's aerial and terrestrial landscapes (Gayk and Mennill 2020). These previous observations suggest that a large proportion of individuals forage and rest in arboreal vegetation and interact directly with terrestrial landscapes (Gayk and Mennill 2020). However, landscape use has not been quantified at a broad geographic scale.

Many bird species broadcast species‐specific calls during migration (Evans and O'Brien 2002; Farnsworth 2005). To verify that counts of these calls detected in audio recordings are an accurate proxy for visual counts of individuals, we conducted visual counts simultaneously with acoustic monitoring at a single study site (Bete Grise) by recording all individuals passing over the recorder to the west in the period during which the acoustic recorder was active. The Pearson correlation between visual counts and acoustic detections was 0.934 (Figure S1). Therefore, we used daily acoustic detections as a proxy for combined aerial and terrestrial landscape use by migrating songbirds.

### 2.2 Acoustic Data Collection

We deployed 12 Song Meter (SM) Mini recorders (Wildlife Acoustics Inc.) (Figure 1 and Table S1) in five seasons: Spring 2022, Fall 2022, Spring 2023, Spring 2024, and Fall 2024 (recording 11,840 h in total). In spring, each device recorded from late April to mid June for 6 h each morning (6 am–noon). In fall, each device recorded from mid August to early October for 6 h (6 am–noon). We chose this morning period to capture morning movements across the peninsula, when low‐flying migrants use and interact with terrestrial landscapes and select locations for stopover (Wiedner et al. 1992; Ball 1952). We chose these 12 sites because they were either known concentration points or close to the Lake Superior coastline. We carefully chose our 12 acoustic monitoring sites to cover as much as possible of the Keweenaw peninsula's coastline landscapes, as prior migratory movement at a few known concentration sites had only been observed in coastal environments. In addition, we included two inland sites in order to sample inland movement.

In 2024 only, we added six additional inland recording sites (Figure 1), which we used for an exploratory analysis comparing inland and coastal locations. The six inland sites were oriented along open inland ridges in an approximate north–south line north of the Bete Grise site. We therefore believe that our focus on capturing the coastal regions where high‐volume movement was known to occur, coupled with our 2024 scheme sampling inland movement, allowed us to accurately assess broad patterns of movement through the Keweenaw peninsula.

We chose to use passive acoustic monitoring in this study to investigate patterns of migratory bird landscape use, as other large‐scale data sources (i.e., citizen‐science eBird data) were not sufficient to capture migration on a landscape scale in the remote focal region of the Keweenaw peninsula. In contrast, acoustic monitoring allowed us to capture entire spring and fall migrations in multiple sites simultaneously using standardized recording schedules.

### 2.3 Acoustic Model Parameter Adjustment

#### 2.3.1 Acoustic Model Training

We used the *Nighthawk* machine learning classifier (Van Doren et al. 2024) to detect and classify putative songbird flight calls in our acoustic dataset. We trained a custom “fine‐tuned” version of the model on a subset of our dataset to improve model performance following the custom batch‐construction technique described in Van Doren et al. (2024). This fine‐tuning procedure consisted of randomly sampling 40 h of audio from each season during spring and fall 2022–2023 and annotating all occurrences of flight calls. We used 50% of these annotations for fine‐tuning and 50% for evaluation. During model training, half of each batch was composed of focal annotations from our dataset; the other half of the batch was composed of annotations from the core Nighthawk training dataset, as described in Van Doren et al. (2024). For identification of flight calls, we used Evans and O'Brien (2002) as a reference. We manually annotated background noises such as frogs, tree noises, and wind and sampled additional background noise annotations for model training. We then trained a custom version of *Nighthawk* that incorporated these locally annotated data to improve model performance on the full dataset.

#### 2.3.2 Initial Model Output

We initially retained all detections from the 12 sites above a score threshold of 0.50, totaling 3.3 million detections. We manually reviewed a random sample of 200 detections per season from each of our focal taxa of Passeriformes, Parulidae, Passerellidae, and Turdidae (a total of 800 detections reviewed each season), assigning each as correct, incorrect, or uncertain. Correct and incorrect assignments were used to calculate precision metrics for each class; uncertain assignments were not used to calculate the precision metrics. We used these data to identify taxon‐specific score thresholds that would achieve a target precision of 0.95 on each class, and we retained all detections above these thresholds (Wood and Kahl 2024). In total, we retained 2,978,474 detections (1,773,767 Passeriformes, 882,841 Parulidae, 290,178 Passerellidae, and 31,688 Turdidae; Table S2).

#### 2.3.3 Radar and Weather Data

We used the ERA5 weather database on single measurement heights (Hersbach et al. 2023) to collect wind data for a grid covering the Keweenaw peninsula and offshore waters of Lake Superior (48 N, −88.2 W, 47.1 S, −86.7 E). We downloaded yearly data for the months of April–November in NetCDF (network Common Data Form) format, summarizing mean wind speed and wind gust data for east–west and north–south winds at 10 m and 100 m above the surface using the tidyverse package in R (R core team 2023; version 2023.1.402; Wickham et al. 2019).

Because the relationship between wind conditions and morning migration may change through the nocturnal and diurnal periods, we calculated the mean and maximum wind vectors for three biologically significant time periods: 9 pm to midnight (early night), midnight to 5 am (late night), and 6 am to 11 am (morning movements). Additionally, to quantify short‐term shifts in wind conditions, we calculated how wind changed from the night to morning by subtracting the wind value between each time period. We calculated the change in wind vectors from night to morning by subtracting the difference in wind vectors from the nocturnal (midnight to 5 am) and morning (6 am to 11 am) periods. We reasoned that migrants crossing Lake Superior might abort their flights and reorient to the Keweenaw Peninsula when winds changed from favorable to unfavorable.

The intensity of nightly bird migration in the study region provides important context for morning movements observed on the Keweenaw peninsula. For example, we generally predicted that landscapes in the Keweenaw peninsula would experience greater usage by migratory birds following larger regional nocturnal migration events. To quantify regional migration intensity, we collected Doppler weather radar data from a nearby NEXRAD (Next Generation Weather Radar) radar station for the duration of the project. The station was KMQT (Marquette, Michigan, located 100.1 km SE of the study area). We used the vol2bird algorithm to extract vertical profiles of speed, direction and density of migrating birds during nocturnal migration. This algorithm is available in the R packages bioRad and vol2birdR (Dokter et al. 2011; Dokter et al. 2019). During data processing, we used the built‐in MistNet model to remove meteorological signals, which can otherwise bias estimates of birds (Lin et al. 2019), and we constructed vertical profiles using only elevation scans cleaned by MistNet (0.5, 1.5, 2.5, 3.5, 4.5 degree sweeps). We used sample volumes within a 5–35 km range from the radar. We applied static beam blockage maps as described in Dokter et al. (2019) and a dynamic clutter mask to filter out anomalous beam propagation and ground clutter. We summarized nightly migration traffic using BioRad (Dokter et al. 2019), restricting analyses to nocturnal periods and using the *integrate_profile* function to integrate across altitudes sampled by the radar. We log‐transformed migration traffic values to reduce skewness, adding a small constant epsilon (*ε* = 1 × 10^−6^) to eliminate negative infinity values (a small value relative to orders‐of‐magnitude larger migration traffic values).

#### 2.3.4 Landscape‐Scale Migration Patterns

To characterize daily patterns of landscape use in our study area, we conducted a Principal Component Analysis (PCA) on daily totals of songbird (Passeriformes) acoustic detections across our 12 primary sites. We used the R package Factoextra (Kassambara and Mundt 2020) to conduct the PCA separately for spring and fall. We repeated this procedure for daily Parulidae, Passerellidae, and Turdidae acoustic detections. We extracted the first three principal components, which accounted for a cumulative 77.8%–78.3% of the variation in flight call detections of Passeriformes. For three sites missing data in spring 2023, we used a data imputation procedure from the R package missMDA (Josse and Husson 2016) during the PCA. For these three sites, data were imputed for the entire spring season in 2023. To aid interpretation of the resulting components, we calculated and visualized correlations between the daily call counts at all sites and daily PC scores for PC1, PC2, and PC3.

The first principal component (PC1) captured overall migrant landscape use across the study area, explaining 84.7% and 84.6% of daily call counts in spring and fall, respectively. Hereafter, we refer to this component as “landscape use intensity.” This component showed medium to high positive correlations across all study sites (Figures S2A, S3A, S4, S7). In other words, high values of PC1 were indicative of days with more migrants present across the entire study area. Although we focused mainly on Passeriformes detections overall, these patterns were generally consistent across Parulidae, Passerellidae, and Turdidae as well.

The second principal component (PC2) captured a distinct directional gradient in migrant landscape use across the study area, explaining 12.1% (spring) and 6.1% (fall) of variation. Specifically, in spring, PC2 was associated with a northwest‐to‐southeast gradient in landscape use (Figures S2B and S5), wherein northern and western sites experienced greater movements than the rest of the study area, or vice versa. High values of PC2 in spring were indicative of days when relatively more birds occurred at northern and western sites compared to the rest of the study area. In fall, PC2 was associated with a west‐to‐east gradient in landscape use (Figures S3B and S8), wherein western sites experienced greater movements than the rest of the study area, or vice versa. An exception was Manitou Island, an island site off the eastern edge of the peninsula, which had low‐to‐moderate positive correlations alongside western sites. Thus, high values of PC2 in fall were indicative of days when relatively more birds occurred at western sites and Manitou Island compared to the rest of the study area.

The third principal component (PC3) captured latitudinal variation in migrant landscape use across the study area and explained 8.2% (spring) and 4.5% (fall) of variation. In both spring and fall, PC3 was associated with a north‐to‐south (latitudinal) gradient of landscape use (Figures S2C, S3C, S6, S9). This component showed high positive correlations with observations along the north shore of the Keweenaw peninsula, but weak or high negative correlations in the south. Thus, high values of PC3 in both spring and fall were indicative of days when more birds occurred at northern sites (including Manitou Island) relative to the rest of the peninsula.

Projecting the six inland and coastal sites only present in spring 2024 onto these principal components showed space usage patterns generally consistent with the primary 12 stations (Figure S10). To visualize broad patterns of migratory landscape use across the Keweenaw peninsula, we produced figures representing the total counts of migrants at each station, and figures representing patterns of landscape use detected in the PCA with the ggmap R package (Kahle and Wickham 2013).

#### 2.3.5 Model Analysis

We used the *gam* function in the R package mgcv (Wood 2011) to construct generalized additive mixed‐effect models examining whether landscape use patterns (quantified by PCs 1–3) were associated with different atmospheric conditions separately for spring and fall seasons, pooling data across years. A gamma distribution was used for all terms in models, except smooth terms, which were specified with *k* = 10. The gam.check function was used to assess model fit by evaluating histograms of the residuals and examining residuals vs. fitted values plots. The response variables in our models were PC1, PC2, and PC3, representing different axes of landscape use. Predictors were the following five wind variables: mean east–west wind, mean north–south wind, AM change in east–west wind, AM change in north–south wind, and radar‐derived nocturnal migration intensity. Date of season was specified as a smooth term in all models to capture expected seasonal variation in migration intensity. For each model, we also included four interaction terms: mean east–west wind × mean north–south wind; mean east–west wind × AM change in north–south wind; mean east–west wind × nocturnal migration traffic; and mean north–south wind × nocturnal migration traffic. We ran models for songbirds overall (order Passeriformes), as well as separately for families Parulidae, Passerellidae, and Turdidae (Table S3).

To identify the most appropriate temporal window for analyses, we conducted an initial analysis using AICc to compare models constructed with atmospheric data from the three different time periods (9 pm to midnight, midnight to 5 am, and 6 am to 11 am) using MuMIn (Barton 2024) and lme4 (Bates et al. 2015) R packages. The candidate model set consisted of one model for each of these time periods. The model with the lowest AICc score used atmospheric data between midnight and 5 am; we therefore used this time period for all further analyses (Table S4).

In spring 2024, we ran an additional analysis using the procedure described above but with the addition of six new inland and coastal sites only present during that year. The objective of this analysis was to determine whether patterns of migrant landscape use were consistent between inland and coastal sites.

## 3 Results

### 3.1 Spatial Patterns of Migrants' Aerial and Terrestrial Landscape Use in the Keweenaw Peninsula

**FIGURE 2:**
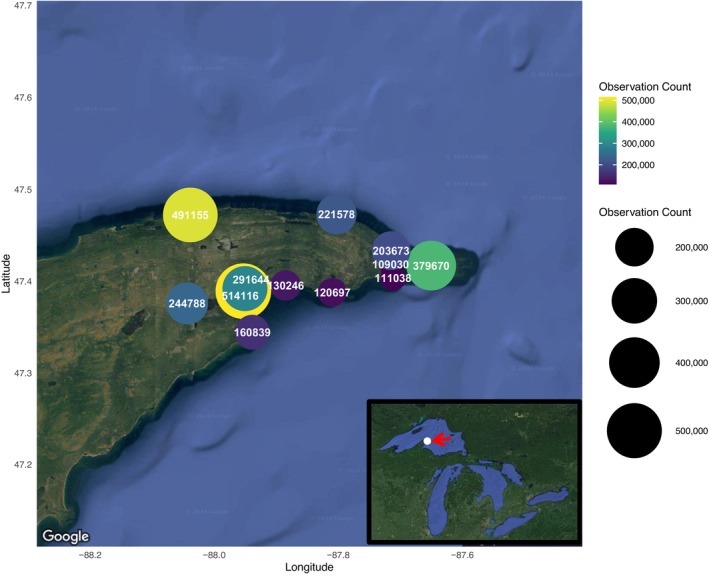
Total flight call detections of migratory Passeriformes engaged in diurnal reoriented migration events at 12 sites in the Keweenaw peninsula, Michigan, USA. The total flight call counts per site in spring 2022–fall 2024 are listed next to each site in white font (only for 12 original sites with multi‐season data shown in red in Figure 1); counts are also colored and scaled according to the relative size of the movement at each site. Maps produced via ggmap R package, with permission from Google.

**FIGURE 3:**
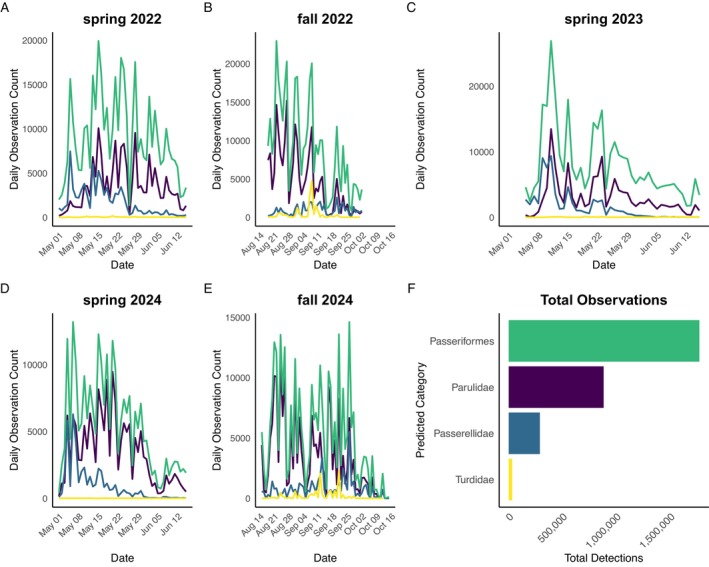
Phenology of acoustic flight call detections of Passeriformes during diurnal reoriented migration events in the Keweenaw peninsula, Michigan, USA. Panels (A–E) provide seasonal time series; green indicates total counts of Passeriformes, purple indicates Parulidae detations, blue indicates Passerellidae detections, and yellow indicates Turdidate detections. (F) shows total detections across taxonomic groups.

In 11,840 h of field recordings, we detected approximately 2.9 million songbird flight calls belonging to order Passeriformes and families Parulidae, Passerellidae and Turdidae (Figure 2 and Table S2). The timing of migration events varied widely across each recording season (Figure 3A–E). There was an uneven distribution of flight call detections between sites, with three sites recording over 300,000 flight call detections (Figure 2). We detected the largest numbers of migrant songbirds over Manitou Island and the coastline of Bete Grise on the south shore, both of which are either on an east–west flight path or at the junction of an east–west and north–south shoreline (Bete Grise; Figure 2). On the north shore, migrant songbirds most heavily used aerial and terrestrial landscapes along the Agate Harbor peninsula, followed by Horseshoe Harbor both overall and in spring (Figure 2 and Figure S11). In fall, larger numbers of migrants were detected at the south shore, such as Bete Grise; fewer migrants were detected at north shore sites in fall (Figure S12).

Our principal components analysis revealed three dominant regimes of landscape use. Usage patterns were generally positively correlated across all sites, as PC1 explained 84.7% and 84.6% of variation in detections in spring and fall, respectively. PC2 and PC3 respectively described additional latitudinal and longitudinal patterns of landscape use across the peninsula. Our supplementary analysis of six inland sites only present in spring 2024 showed space usage patterns generally consistent with the primary 12 stations (Figure S10).

### 3.2 Landscape Use Intensity and Lake Superior Wind Patterns

**FIGURE 4:**
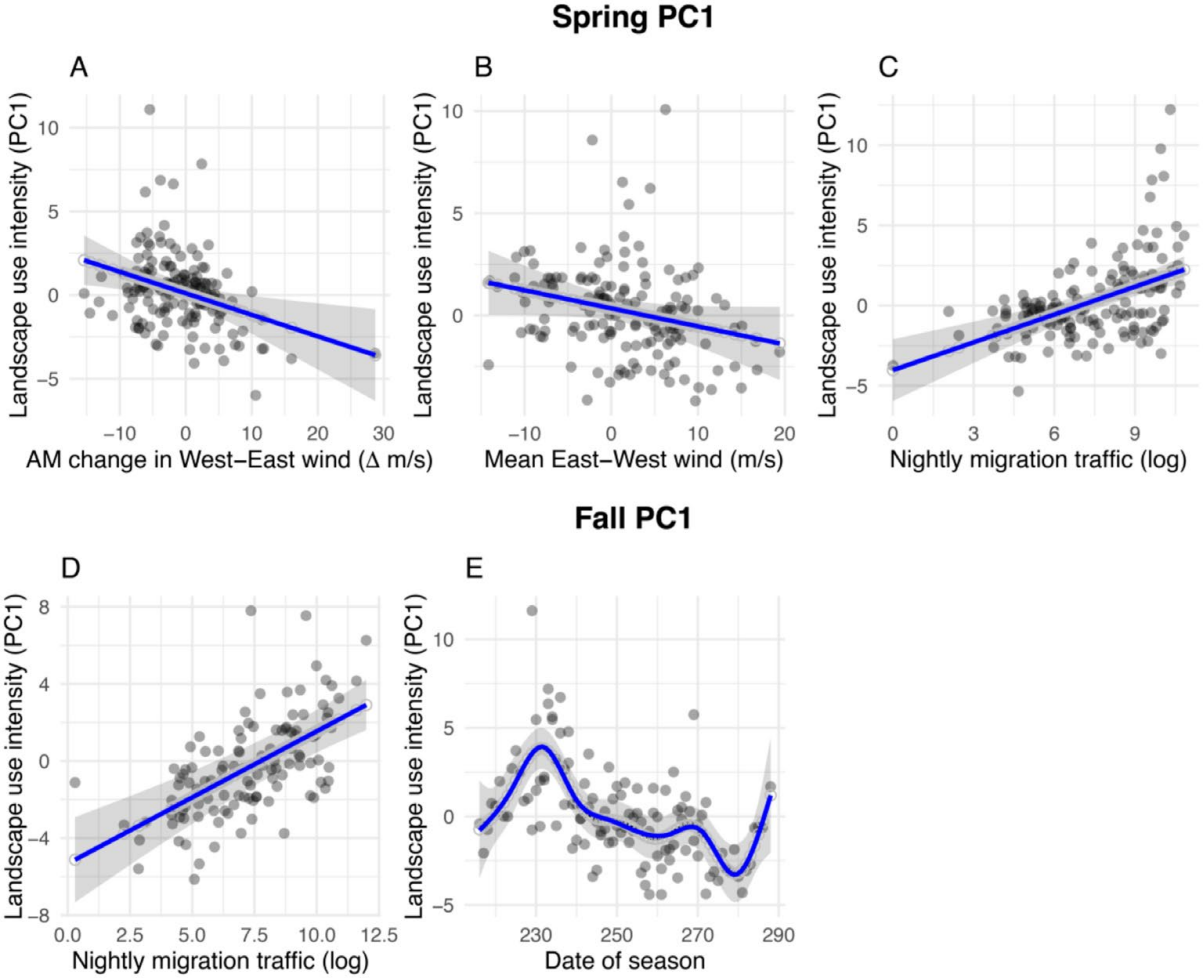
PC1. Relationships of landscape use intensity during diurnal reoriented migration of Passeriformes in the Keweenaw peninsula, Michigan during spring (top panel) and fall (bottom panel). Each plot represents model predictions based on partial residuals for (A) Relationship between change in west–east wind over Lake Superior between night and morning and overall landscape use intensity of Passeriformes migration (spring), (B) Overall east–west wind over Lake Superior and landscape use intensity of Passeriformes migration (spring), (C) Relationship between nightly migration traffic over Lake Superior and overall landscape use intensity of Passeriformes (spring), (D) Relationship between nightly migration traffic over Lake Superior and overall landscape use intensity of Passeriformes (fall), (E) Date of season and overall landscape use intensity of Passeriformes (fall). X‐axis labels indicate direction of wind with (A) indicating negative change in m/s as increasing west wind and positive change in m/s as east wind. Axis labels for (B) indicate negative wind (in m/s) as east wind and positive (in m/s) as west wind. Model predictions based on partial residual contributions were plotted using the *predict_response* function in the sjPlot R package.

Lake Superior wind patterns significantly predicted overall migrant landscape use across the Keweenaw peninsula during spring. In spring, we observed important effects of shifting winds: use of the peninsula increased when offshore winds increased in strength from the west during the early morning (Figure 4A). The offshore east–west wind component between midnight and 5 am also predicted use: migrants used peninsula landscapes more heavily when offshore winds were from the east between midnight and 5 am (Figure 4B). Nocturnal migration traffic also predicted overall migrant landscape use across the Keweenaw peninsula in both spring and fall: more intense nocturnal migration events resulted in greater use of the peninsula (PC1) during the following morning (Table S5 and Figure 4C,D).

In fall, wind did not strongly predict landscape use, although usage patterns varied consistently through the fall season, becoming most positive in the early and middle parts of the migration season. This suggests that more birds were detected overall in the peak of the migratory season for Passeriformes (Table S5 and Figure 4E).

### 3.3 Northwest‐To‐Southeast Gradients of Landscape Use

**FIGURE 5:**
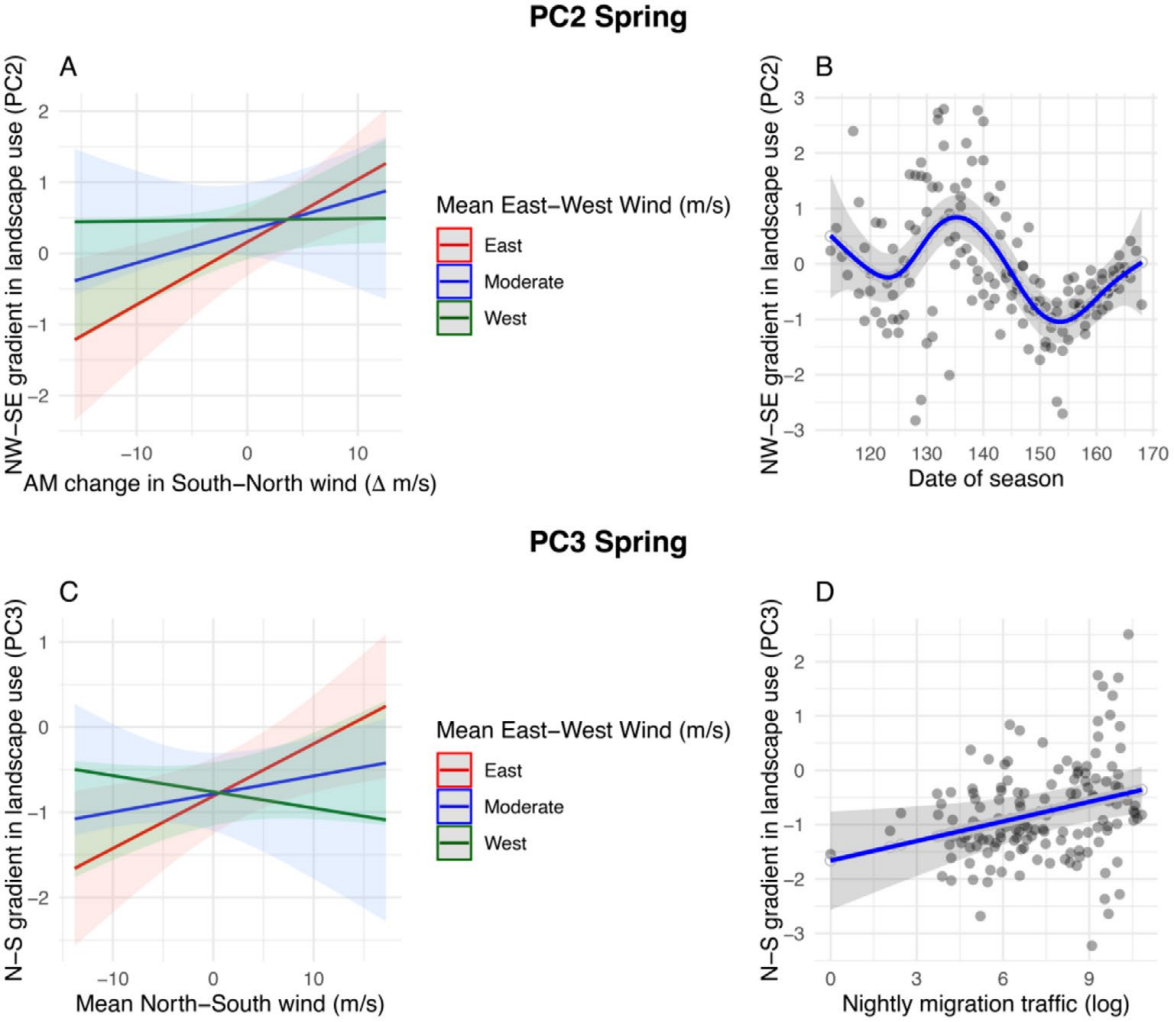
PC2 and PC3 spring. Relationships of northwest‐to‐southeast gradient in landscape use intensity during diurnal reoriented migration (PC2) of Passeriformes in the Keweenaw peninsula, Michigan during spring (top panel). Each plot represents model predictions based on partial residuals for (A) Interaction of morning change in south–north wind over Lake Superior with overall east–west wind over Lake Superior and northwest‐to‐southeast gradient in landscape use intensity of Passeriformes migration. (B) Date of season and northwest‐to‐southeast gradient in landscape use intensity of Passeriformes migration (top panel). The bottom panel (PC3) represents relationships between weather and latitudinal variation in landscape use intensity during diurnal migration in the Keweenaw peninsula during spring. Each plot represents model predictions based on partial residuals for (C) Interaction of mean north–south with east–west wind over Lake Superior and latitudinal variation in landscape use intensity. (D) Nightly migration traffic over Lake Superior and latitudinal variation in landscape use intensity. X‐axis labels indicate direction of wind with (A) indicating negative change in m/s as increasing south wind and positive change in m/s as increasing north wind. Axis labels for (C) indicate negative wind (in m/s) as north wind and positive (in m/s) as south wind. Model predictions based on partial residual contributions were plotted using the *predict_response* function in the sjPlot R package.

Wind patterns significantly influenced spatial variation in songbird landscape use on a northwest‐to‐southeast gradient (PC2) across the Keweenaw peninsula (Table S5). In spring, overnight shifts in offshore north–south winds predicted subsequent landscape use, modulated by offshore east–west winds. Specifically, higher landscape use intensity occurred in the northwestern region relative to the rest of the peninsula when winds were increasing from the north in the morning while simultaneously from the east (Figure 5A). These landscape use patterns also varied consistently through the spring season, with observed values of PC2 becoming most positive in the middle of the migration season. This suggests that more birds were detected in the northwestern region in the peak of the migratory season (Figure 5B). In contrast, in fall, we observed no effects of wind on this axis of landscape use. Nocturnal migration intensity did not influence this axis in either season.

### 3.4 Latitudinal Patterns of Landscape Use

Wind patterns significantly influenced spatial variation in landscape use on a latitudinal gradient (PC3) across the Keweenaw peninsula (Table S5). In spring, offshore north–south winds between midnight and 5 am predicted the latitudinal gradient in diurnal migration intensity, modulated by offshore east–west winds (Table S5 and Figure 5C). Specifically, we recorded higher landscape use intensity in the north of the peninsula when winds were from the south while simultaneously from the east (Figure 5C). Latitudinal patterns of landscape use also varied through the spring season, with observed values of PC3 becoming more positive in the middle of the migration season (relationship nearly identical to that shown in Figure 5B; see Table S5). This suggests more birds were detected in the northern coastal region in the peak of the migratory season (Table S5). Landscape use was relatively higher in the north of the peninsula following spring nights with more intense nocturnal bird migration (Table S5 and Figure 5D). In contrast, in fall, we observed no effects of wind or nocturnal bird migration intensity on this axis of landscape use.

## 4 Discussion

Our results demonstrate that migratory birds' landscape use is shaped by geographic features of land and water, particularly as birds reorient following unfavorable migratory conditions over the Lake Superior barrier. We found that northbound songbirds in spring use the Keweenaw peninsula following morning westerly, nocturnal easterly, and morning northerly winds over Lake Superior, primarily concentrating along east–west oriented coastal and ridge landscapes. These results suggest that migratory songbirds' spring routes are subject to alteration (Kürten et al. 2025) if weather conditions are not conducive to crossing the Lake Superior barrier. We also found that overall landscape use intensity at this crossing is closely linked to regional migration traffic in both spring and fall, indicating that the Keweenaw peninsula represents a regionally important site for migrating songbirds. Together, our results illustrate the complex role large water barriers play in shaping broad geographic patterns of landscape use by highly mobile animals such as migratory songbirds.

### 4.1 Migratory Landscape Use Patterns in the Keweenaw Peninsula

Our results indicate that reorienting songbirds are heavily concentrated by both Lake Superior and geographic features of the Lake Superior coastline, a result consistent with studies in Quebec and Cape May, New Jersey (Wiedner et al. 1992). In particular, our analyses identified several hotspots of landscape use across the Keweenaw peninsula. We recorded > 300,000 detections at three sites: (1) Manitou Island, off the eastern tip of the peninsula; (2) Agate Harbor, a narrow peninsula on the north shore; and (3) the Bete Grise shoreline, at a marked geographic junction on the south shore. At these locations, geographic features cause migrants moving west through the landscape to concentrate at extremely high densities.

The orientation of the coastline had a strong effect on bird behavior, especially when oriented in a direction consistent with migrants' preferred westward direction of movement. We detected the highest total concentrations of migrating songbirds at the Bete Grise site, located at a junction of east–west and north–south oriented coastlines. Intermediate numbers of migrants used sites with an east–west orientation that lacked a change in orientation, or at inland ridge sites parallel to Lake Superior. The lowest numbers of migrants used habitats along north–south oriented coastlines that did not allow migrants to continue westward away from Lake Superior.

We found that the intensity of landscape use tended to be correlated across all of our monitoring sites, which reflects broad coordinated movements across the 328 km^2^ covered by our study. We also found a secondary geographic gradient in landscape use intensity from northwest to southeast, with higher correlations with bird movement in the northwest. This gradient in landscape use patterns may represent migrants' preference for westward movement through the peninsula. We also detected disparate patterns of landscape use across seasons, with a preference for the north shore in spring and the south shore in fall. This contrast may reflect migrants' preferred directions of movement, with most individuals in spring bound for destinations to the north, and vice versa. Overall, these results suggest that the Keweenaw peninsula and coastal regions in general serve important functions for large numbers of migrating birds (Binford 2006; Kaplan and Youngman 2011; Smolinsky et al. 2013). As many migratory species continue to decline, we believe that quantifying regions of high‐intensity landscape use with acoustic monitoring will become a valuable approach to identify important but overlooked regions for migrating birds.

### 4.2 The Influence of Wind and the Role of the Keweenaw Peninsula in Barrier Crossing

We found that wind conditions strongly influenced how migratory birds used landscapes in the Keweenaw peninsula during spring, and that these relationships were inextricably linked to the peninsula's location adjacent to a large water barrier (Archibald et al. 2017). An important but underexplored aspect of migration is how dangerous barriers to migration such as Lake Superior shape migratory behaviors and alter birds' migratory routes under varying weather conditions (Adamík et al. 2016). The decisions that motivate migratory animals' behavior when crossing potentially dangerous barriers are not well understood (Kranstauber et al. 2023), especially those that cause birds engaged in nocturnal migration across the barrier to abort a crossing attempt and initiate reoriented migrations (Lavallée et al. 2021). Further, the conditions that cause birds to initiate reoriented migration events during the day as opposed to continuing nocturnal migration are not well understood (Lavallée et al. 2021; Terrill et al. 2021).

Migratory songbirds using habitats in the Keweenaw peninsula following nocturnal migrations may be doing so for multiple reasons. One hypothesis is that these birds are engaging in diurnal movements through the landscape as a *continuation* of their nocturnal migration, taking advantage of favorable conditions to make further progress toward their destinations (Schmaljohann et al. 2022). Under this hypothesis birds should not deviate from their migratory directions and potentially be composed of migrants with fast transit times (Rüppel et al. 2023). An opposing hypothesis is that birds might continue migration during the day if nocturnal conditions for migration across large barriers, such as Lake Superior, are suboptimal, forcing migrants to reorient to favorable habitat as they are exposed to dangerous over‐water conditions post‐sunrise (Smolinsky et al. 2013).

Our findings are primarily consistent with the latter explanation, especially in spring. Large diurnal migration events along the south shore of Lake Superior appear to represent reoriented migration (Van Doren et al. 2016; Archibald et al. 2017) following attempts to cross the water barrier. Migrants used the Keweenaw peninsula primarily on mornings following heavy migration traffic across Lake Superior while winds over Lake Superior had unfavorable crosswind or headwind vectors. Furthermore, our results are consistent with an important prediction of the reoriented migration hypothesis: that the length and direction of reoriented diurnal migration events should compensate for the degree of wind drift encountered by migrants (Van Doren et al. 2016; Archibald et al. 2017). We found that offshore westerly winds increasing between night and morning were associated with westward diurnal migratory movements of songbirds in spring. In addition, offshore northeasterly headwinds increasing over Lake Superior during morning appeared to be important in driving a northwest–southeast gradient in landscape use, which suggests migrants reorient to land after encountering unfavorable northerly headwinds for crossing the Lake Superior barrier.

We do find some support for the hypothesis that birds using the Keweenaw peninsula may be continuing migration on favorable wind patterns. We observed some large diurnal migration events following southerly tailwinds (in spring) as well as some diurnal migration events following easterly winds, which likely drive birds to the Keweenaw peninsula. For example, we found that a north–south latitudinal gradient in landscape use during spring may be driven by spring southeast winds, offering the opportunity for migrants to harness favorable winds to reach the north shore of the peninsula, consistent with their desired northerly destinations. The relationship between wind and landscape use patterns is likely complex, perhaps reflecting migrants' desires to arrive on breeding ground early coupled with diurnal searches for suitable stopover habitat when conditions for continuing migration over barriers are unfavorable.

Unlike in spring, offshore wind conditions in fall do not appear to explain patterns of migrant landscape use in the Keweenaw peninsula. One possibility is that the Keweenaw peninsula's position along the south shore of Lake Superior means that migrants crossing the lake are more likely to abort crossing attempts in the spring, when the vast bulk of the Lake Superior barrier lies in front of them. In contrast, fall migrants have already crossed most of Lake Superior and therefore may be less likely to reorient to the Keweenaw peninsula on specific unfavorable winds. In fall, landscape use of the Keweenaw peninsula may be influenced more by other factors such as energetics, timing of crossing attempts, and the convenient geographic position of the peninsula as a place to land after crossing the lake.

### 4.3 Implications of Landscape Use Patterns on Conservation

Migratory birds are likely in critical need of stopover habitat for refueling and reorientation after attempting to cross Lake Superior, a time when migrants are very vulnerable. Currently, wind energy prospecting is ongoing for the entire Lake Superior region (Ashtine et al. 2016) and future research should examine migratory pathways (Schmaljohann et al. 2022) and regions of high density for songbirds overall and species of conservation concern. Large concentrations of migrants using habitats of the Keweenaw peninsula for post‐dawn reorientation and morning refueling indicate the importance of this region for migrants.

Although our study region has relatively intact habitats, a rapidly expanding tourism industry has led to numerous development proposals. Future land management planning should be sensitive to high numbers of migratory songbirds migrating over and using terrestrial habitats of the peninsula after reorientation from Lake Superior. As artificial light at night (Horton et al. 2019; Winger et al. 2019) and large glass windows are well demonstrated to disorient migratory birds, leading to fatal collisions, future tourism developments should be careful to minimize risks to migratory birds with careful planning and siting considerations.

### 4.4 Future Directions

Acoustic monitoring is an increasingly valuable tool for studying movement patterns of migratory animals. Although widely used for general biodiversity monitoring (Sugai et al. 2019), as well as general monitoring of migratory birds' night flight calls (Van Doren et al. 2024), acoustic monitoring has been rarely used as a tool to study movement patterns of migrants across the landscape. Understanding patterns of landscape use across broad geographies is a current objective of conservation research (Petit 2000) with the potential to yield novel insight into how migrants distribute themselves heterogeneously over variable environments. Our study demonstrates that acoustic monitoring has broad potential to answer a number of pressing questions about migrant landscape use.

Acoustic monitoring offers the potential to complement tools such as radar (Cohen et al. 2021) and bring new insight on how migrants use important regions of the landscape. New insights could include fine‐scale information on habitat use across the diel cycle and during different seasons, and on distribution patterns in response to landcover and ecological features requiring conservation attention. One question relevant to urban areas is how migrants' landscape use patterns in cities relate to existing greenspace and high‐risk urbanized areas. Acoustic monitoring stations generally have a limited range of a few hundred meters for migratory songbirds (Gayk and Mennill 2020), but we believe that this limitation can be remedied through integrated networks of acoustic monitoring stations in concert with radar. Together, acoustics and radar may allow access to complementary and novel information on how fast‐moving migrants use the landscape.

Future work should use acoustic networks and radar to simultaneously monitor migration at multiple points across Lake Superior, which could reveal novel insights into the distributions of migrants and species during barrier crossings. Although we focus on songbird migration generally, species likely differ in barrier crossing propensity and reorientation behavior. A promising line of inquiry would be to examine how multiple species with different natural history traits such as flight speed, wing loading, and migration distance vary in their reorientation behavior with strength of weather conditions. Research in these areas is likely to produce new information at fine taxonomic scales about how migratory species use habitats across landscapes with various degrees of stopover suitability during migration. An additional area for future research would be to record flight calls during nocturnal migration and examine how the social associations of migrant species differ between nocturnal migration and morning flight. Research in this area offers the potential to reveal new insight into the importance of species associations during migration and whether these associations are stable as migrants transition from onward migration to a search for stopover habitat.

## Author Contributions

**Zach G. Gayk:** conceptualization (equal), formal analysis (equal), investigation (equal), writing – original draft (equal), writing – review and editing (equal). **Benjamin M. Van Doren:** conceptualization (equal), formal analysis (equal), investigation (equal), software (lead), writing – original draft (equal), writing – review and editing (equal).

## Conflicts of Interest

The authors declare no conflicts of interest.

## Supporting information


**Data S1:** ece372635‐sup‐0001‐Supinfo.pdf.

## Data Availability

We include all necessary data and code for the analyses in the paper at the following Dryad repository: https://doi.org/10.5061/dryad.5mkkwh7hq.
