# Supplementary material for: Bioacoustic Monitoring Reveals Patterns of Landscape Use by Migrating Birds at a Great Lakes Barrier Crossing
Source: Ecol Evol. 2025 Dec 16;15(12):e72635. doi: 10.1002/ece3.72635 (PMC12707026; doi:10.1002/ece3.72635)
Supplement: Supplementary file 1 — Data S1: ece372635‐sup‐0001‐Supinfo.pdf. [file ECE3-15-e72635-s001.pdf]

Supplemental Tables and Figures

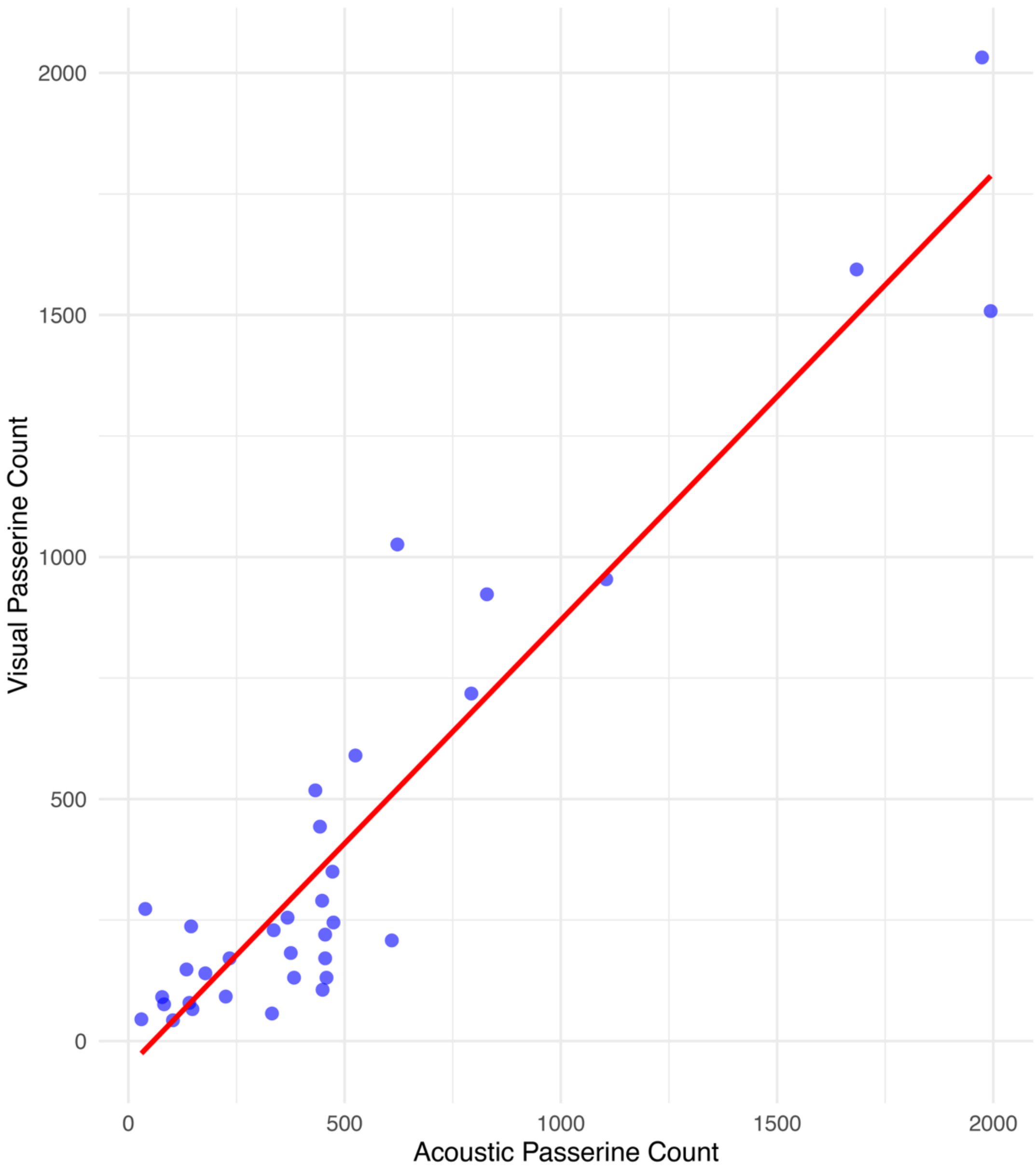

Supplemental Figure 1. Scatterplot of acoustic passerine counts and visual counts of diurnal reoriented songbird migration at the Bete Grise monitoring station in the Keweenaw peninsula, Michigan, USA, from spring 2022–2024. Visual counts were conducted by recording all identifiable migrant Passeriformes travelling west at this location between 6am-12pm each morning. Passeriformes that could not be identified to species were filtered out of this dataset. The overall Pearson correlation between acoustic and visual counts in these seasons was 0.934, while individual correlations for each season were as follows: spring 2022: 0.754, spring 2023: 0.867, spring 2024: 0.600.

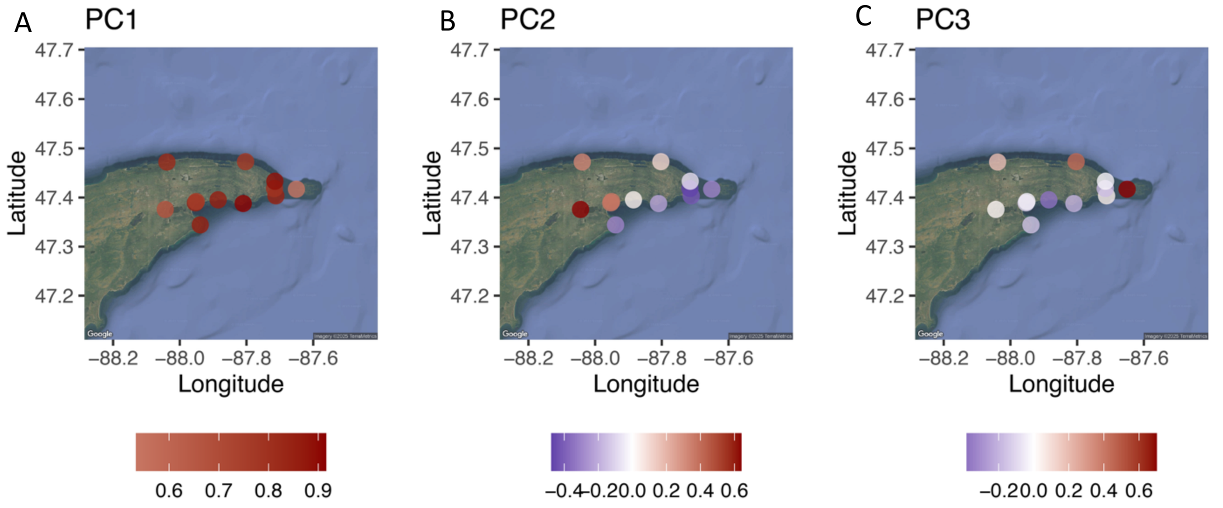

Supplemental Figure 2. Correlations between daily spring Principal Components scores and daily counts of Passeriformes detections at 12 sites in the Keweenaw peninsula, Michigan. A: Overall landscape use intensity during diurnal migration across the Keweenaw peninsula PC1, B: Northwest-to-Southeast gradient in landscape use intensity across the peninsula PC2, C: North-to-South gradient in landscape use intensity across the peninsula PC3.

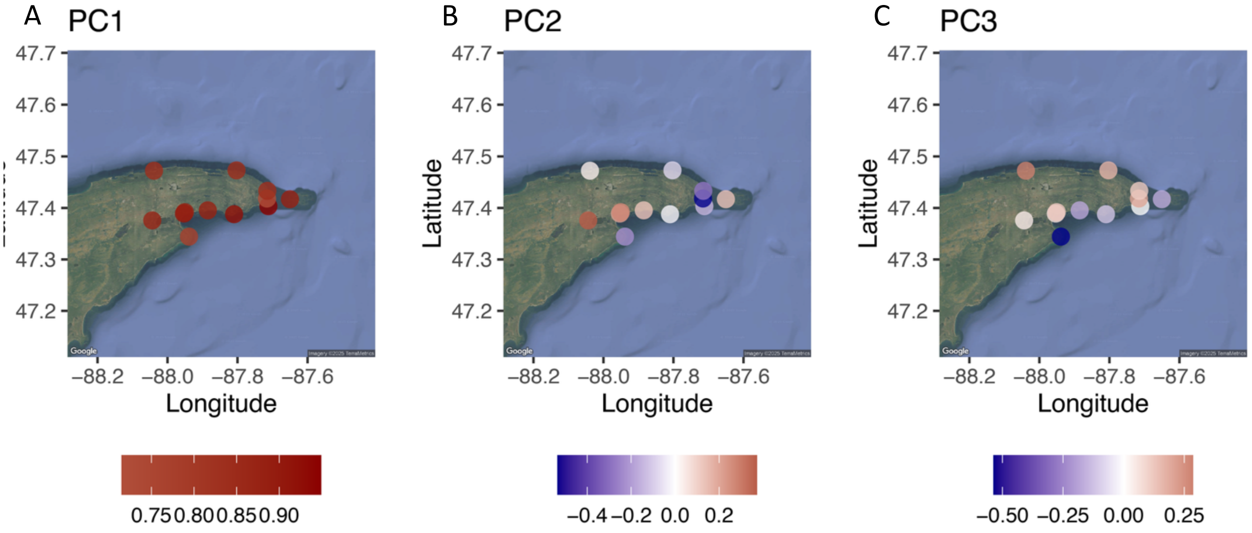

Supplemental Figure 3. Correlations between daily fall Principal Components scores and daily scaled counts of Passeriformes detections at 12 sites in the Keweenaw peninsula, Michigan. A: PC1 which represents overall landscape use intensity during diurnal migration, B: PC2 which represents West-to-East gradient in landscape use intensity, C: PC3 which represents North-to-South gradient in landscape use intensity.

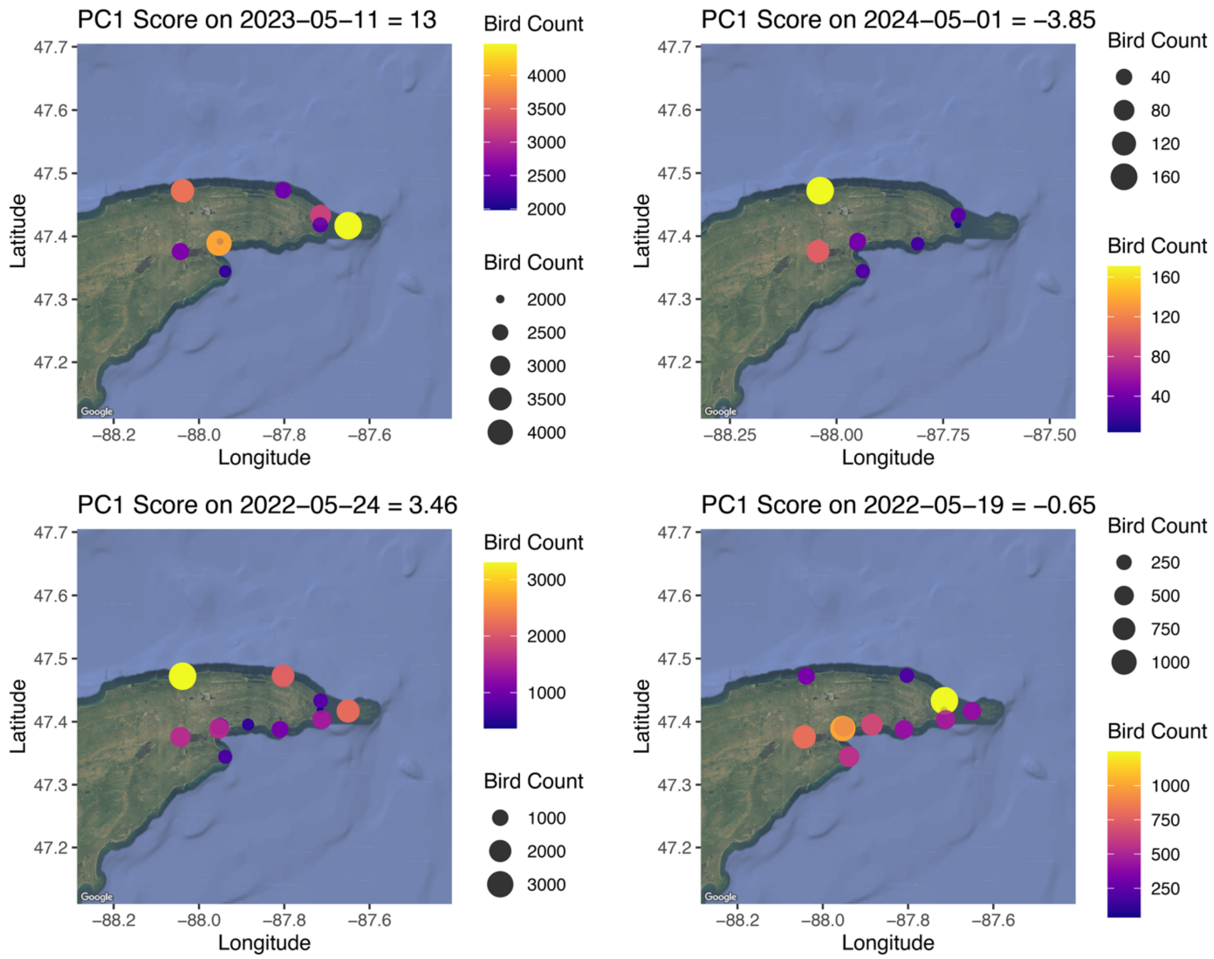

Supplemental Figure 4. PC1 representing high, low and intermediate spring PC scores indicating overall landscape use intensity of diurnal migration in the Keweenaw peninsula, Michigan, USA versus actual bird counts at 12 acoustic recording sites.

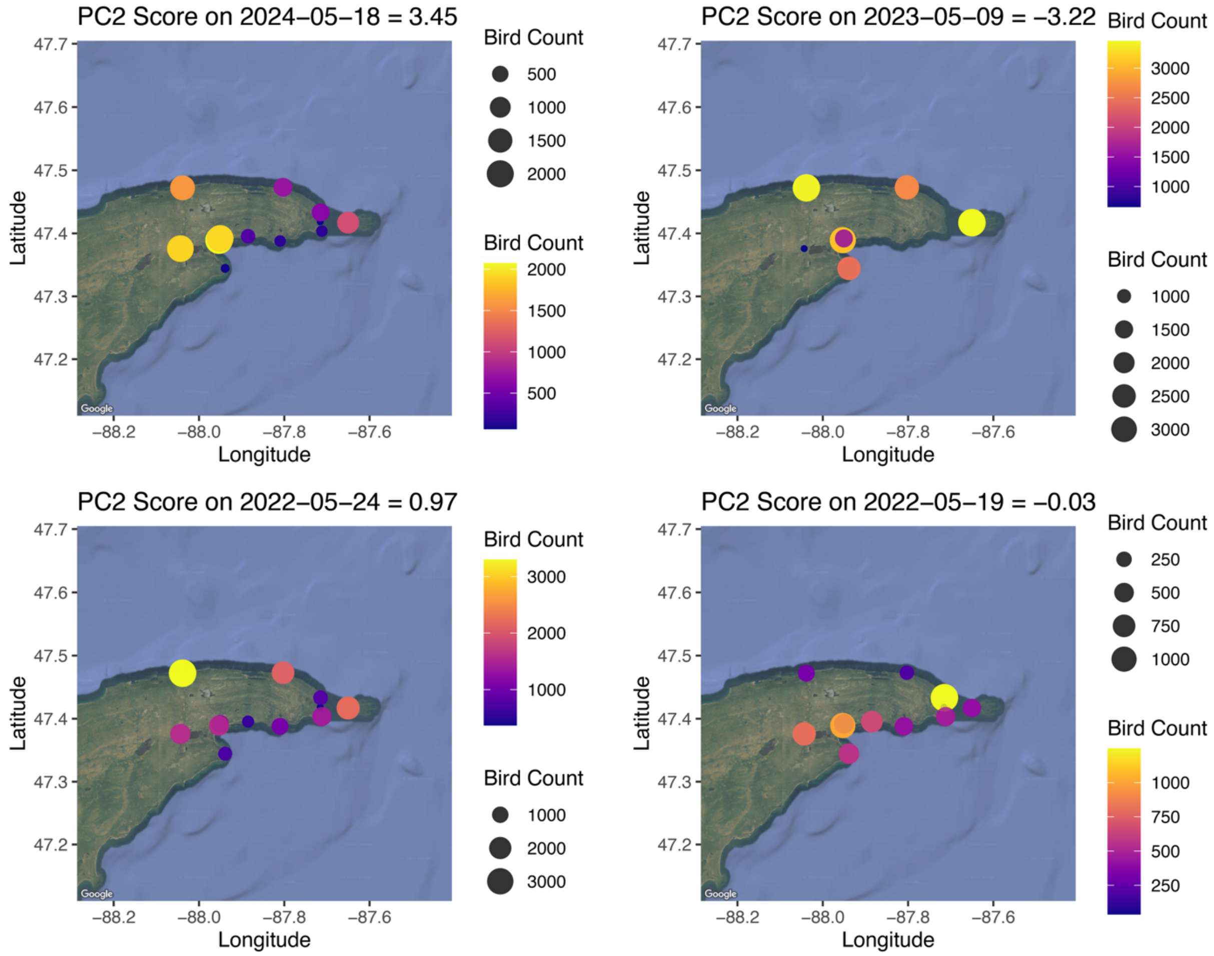

Supplemental Figure 5. PC2 representing high, low and intermediate spring PC scores indicating overall landscape use intensity of diurnal migration in the Keweenaw peninsula, Michigan, USA versus actual bird counts at 12 acoustic recording sites.

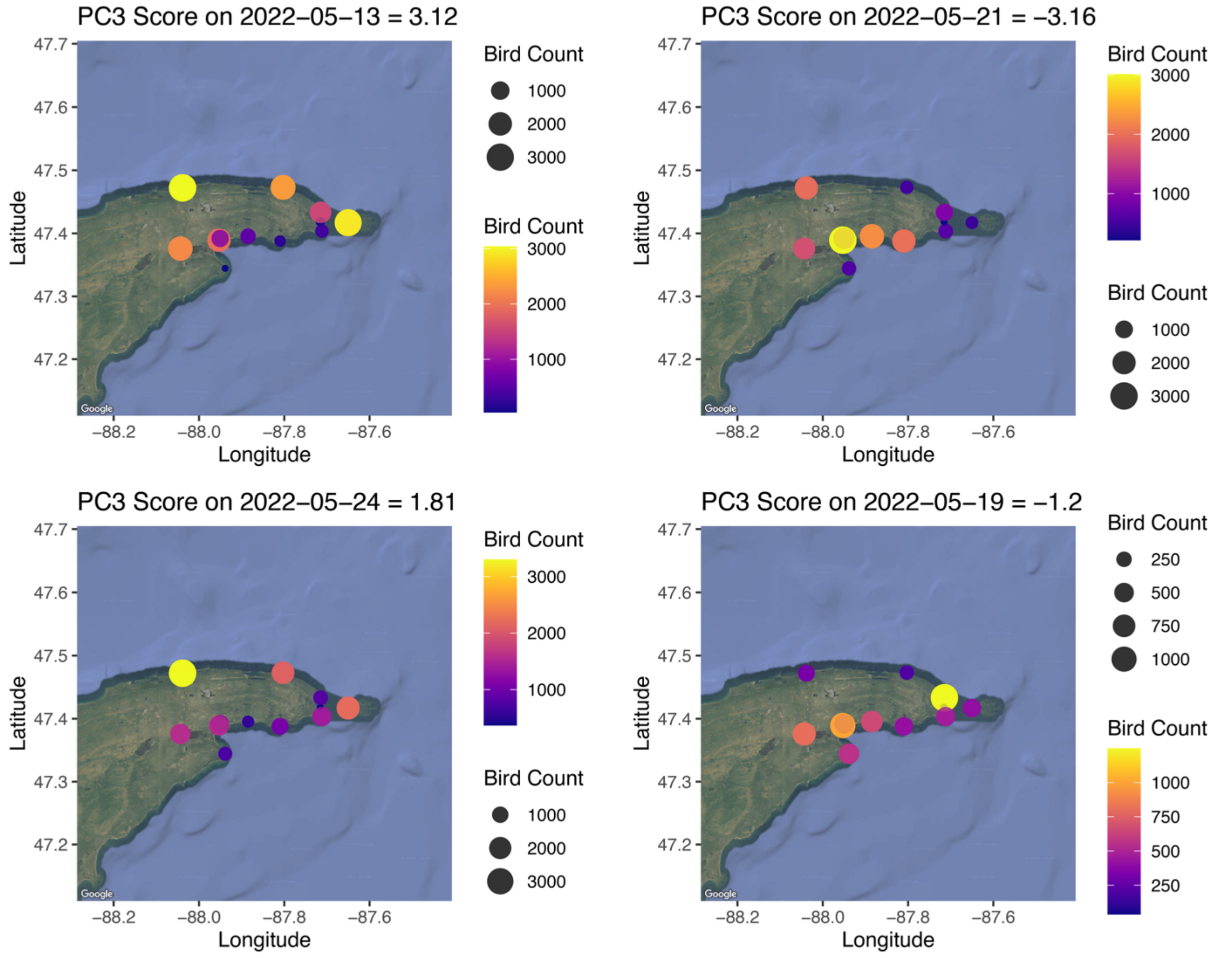

Supplemental Figure 6. PC3 representing high, low and intermediate spring PC scores indicating overall landscape use intensity of diurnal migration in the Keweenaw peninsula, Michigan, USA versus actual bird counts at 12 acoustic recording sites.

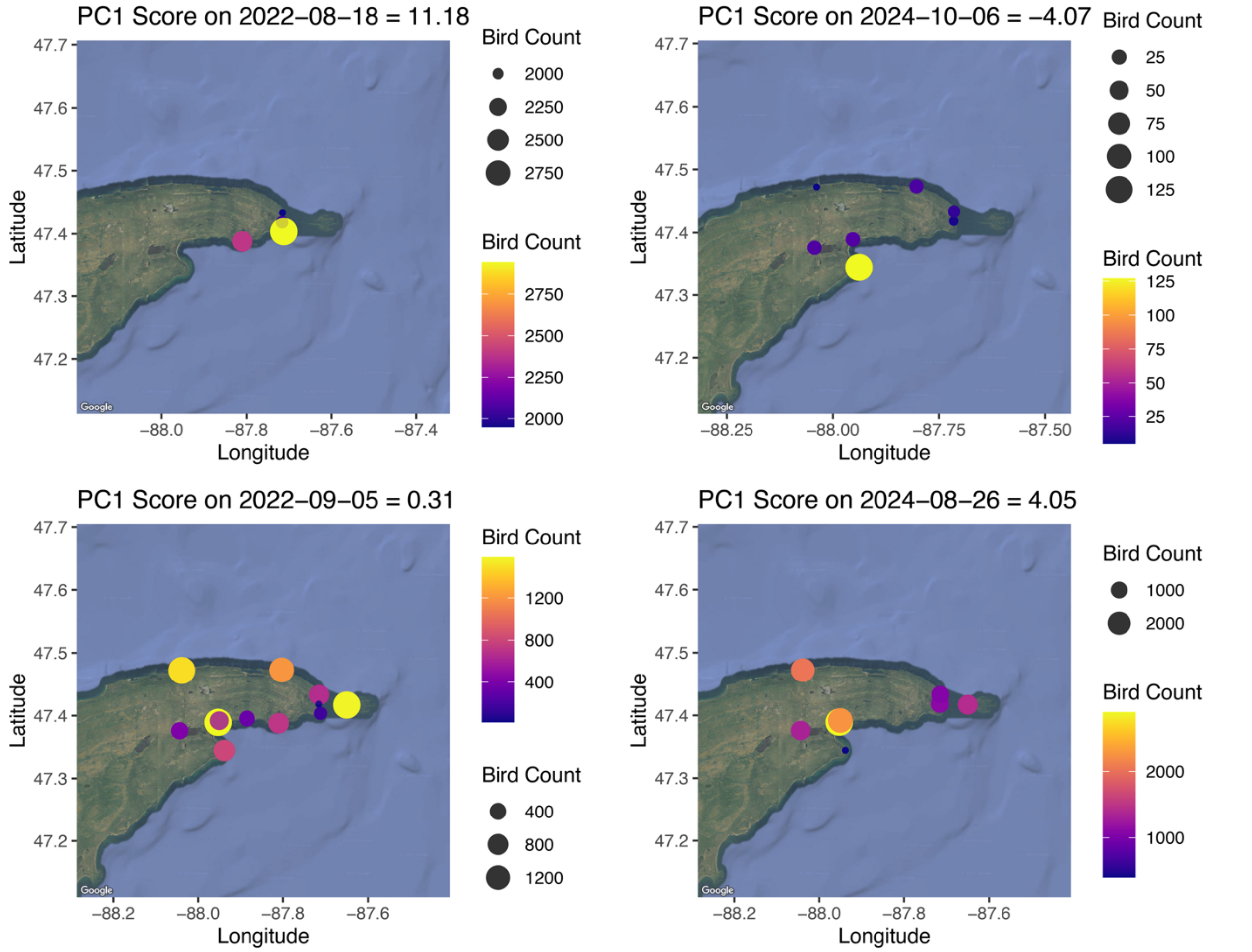

Supplemental Figure 7. PC1 representing high, low and intermediate fall PC scores indicating overall landscape use intensity of diurnal migration in the Keweenaw peninsula, Michigan, USA versus actual bird counts at 12 acoustic recording sites.

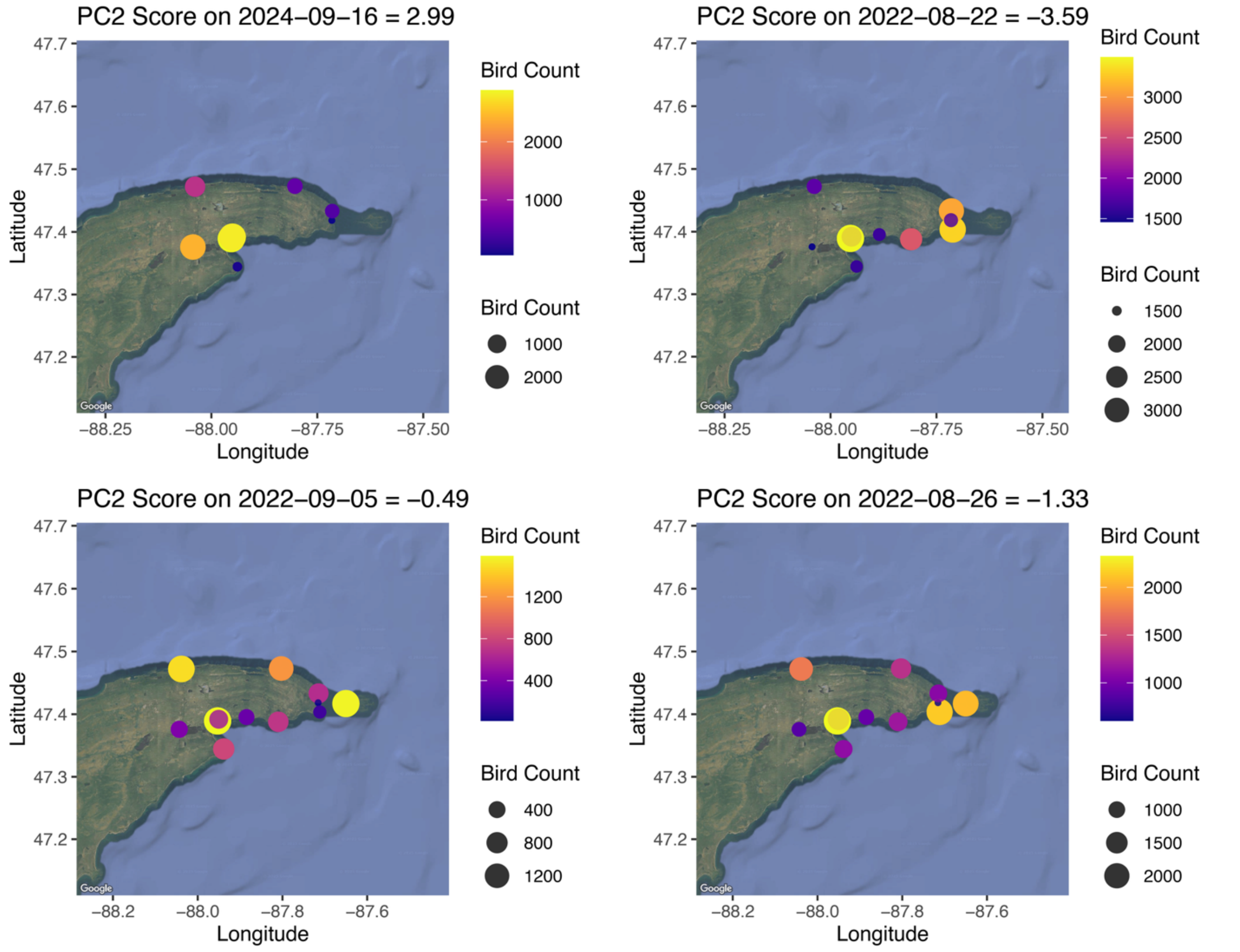

Supplemental Figure 8. PC2 representing high, low and intermediate fall PC scores indicating overall landscape use intensity of diurnal migration in the Keweenaw peninsula, Michigan, USA versus actual bird counts at 12 acoustic recording sites.

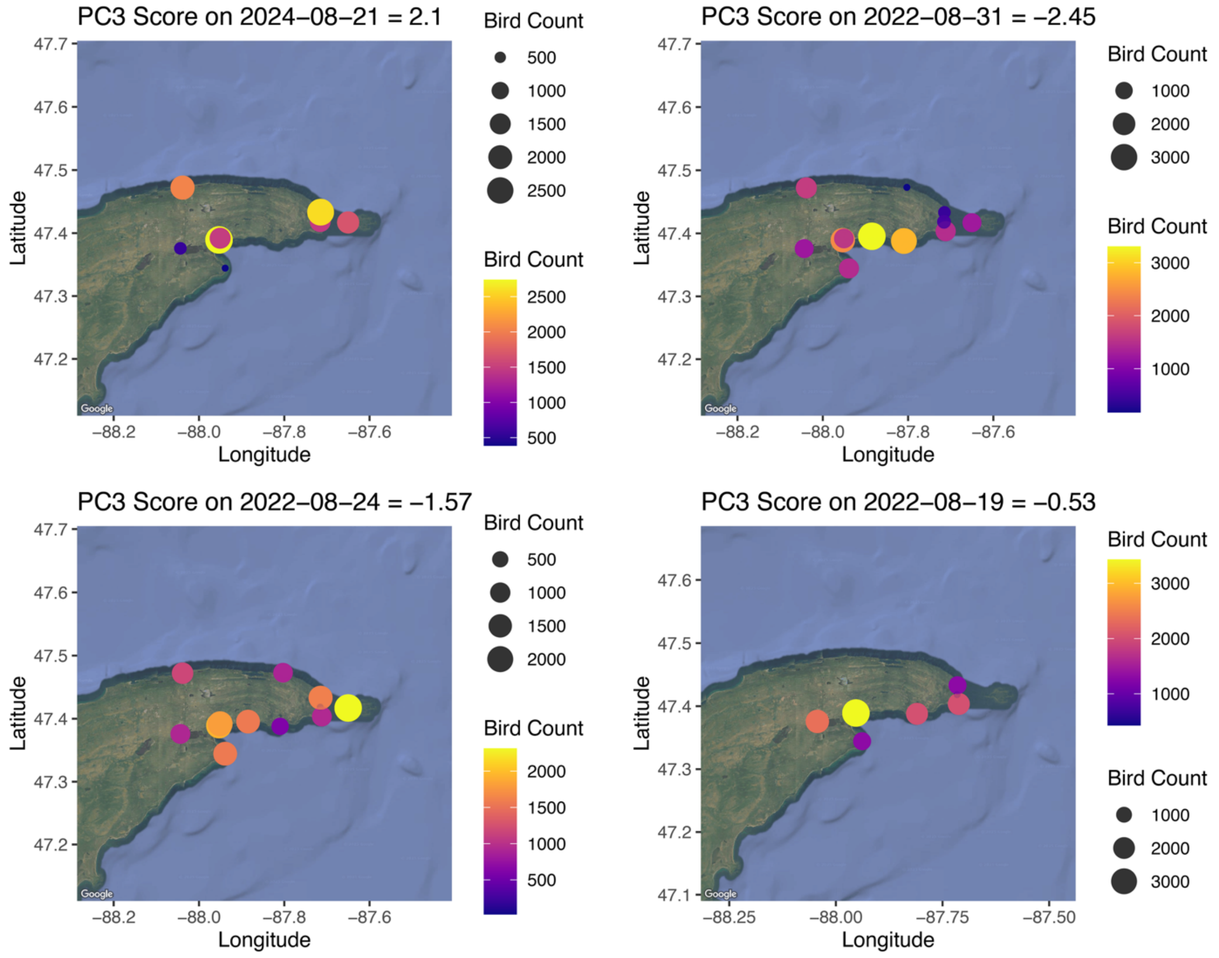

Supplemental Figure 9. PC3 representing high, low and intermediate fall PC scores indicating overall landscape use intensity of diurnal migration in the Keweenaw peninsula, Michigan, USA versus actual bird counts at 12 acoustic recording sites.

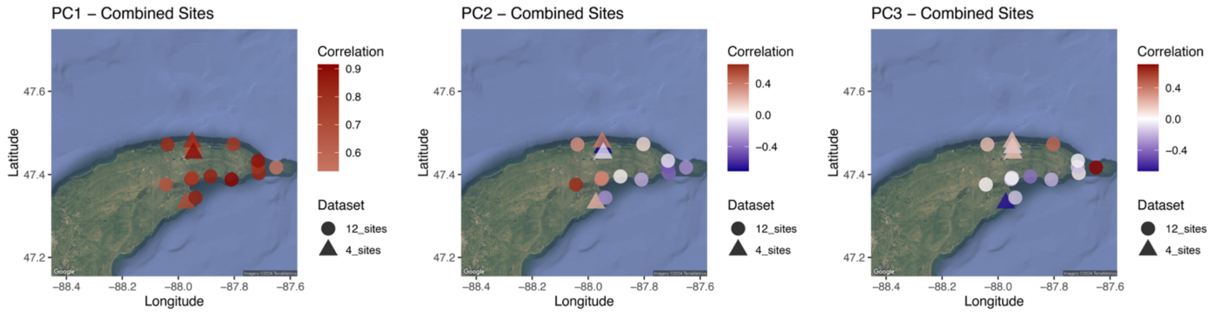

Supplemental Figure 10. Correlations between daily spring Principal Components scores and daily counts of Passeriformes detections at 18 sites in the Keweenaw peninsula, Michigan, with a focus on six inland sites new in 2024. A: Overall landscape use intensity during diurnal migration across the Keweenaw peninsula PC1, B: Northwest-to-Southeast gradient in landscape use intensity across the peninsula PC2, C: North-to-South gradient in landscape use intensity across the peninsula PC3. Correlations between PC scores and bird counts for the original 12 sites are show as circles while correlations between PC scores and bird counts for 4 new sites in 2024 are show as triangles. Space usage patterns at four inland stations were generally consistent with those of the primary 12 stations. One exception was an inland site on the north coast of the peninsula that was negatively correlated with PC2.

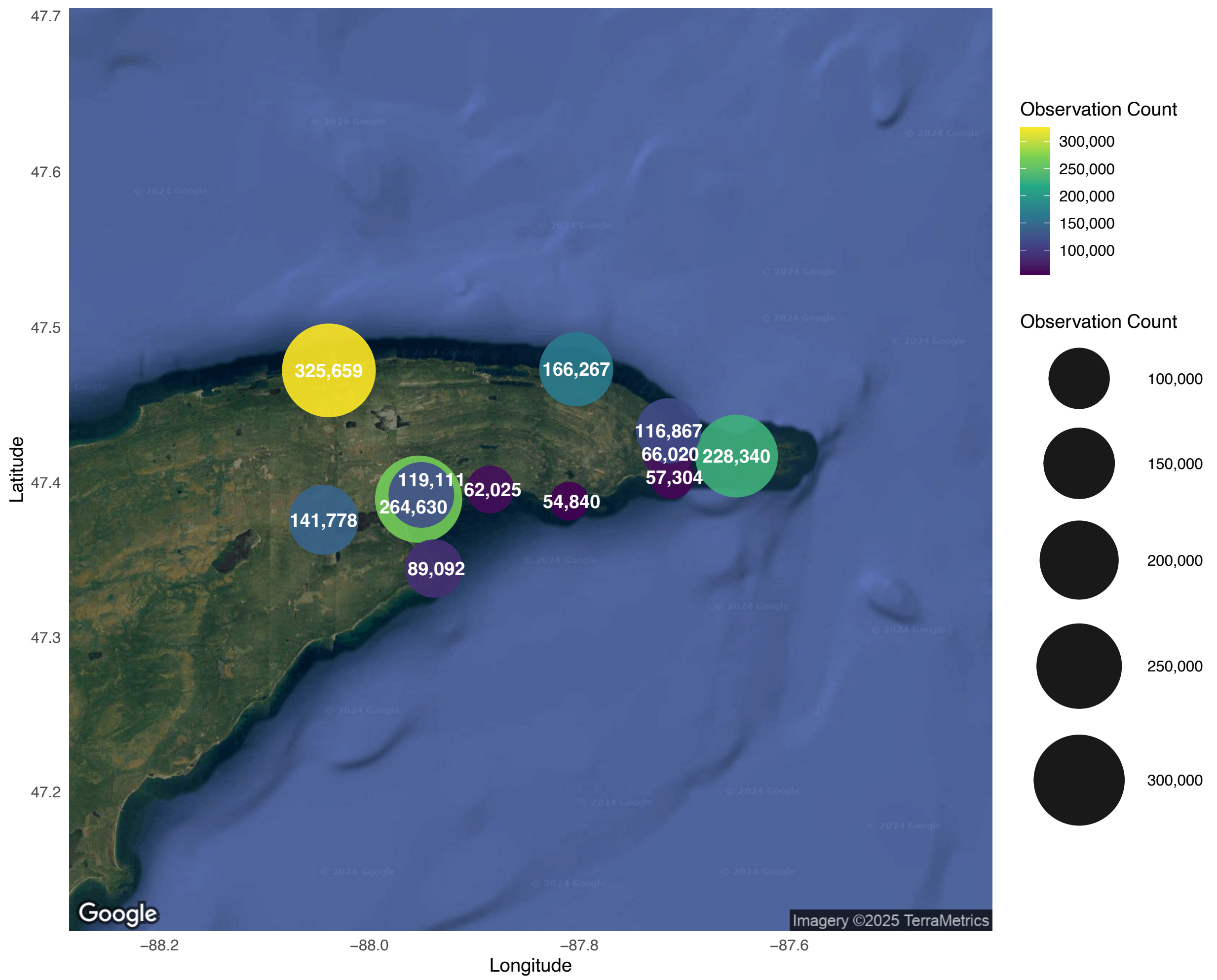

Supplemental Figure 11. Spring flight call detections of migratory Passeriformes engaged in diurnal reoriented migration events at 12 sites in the Keweenaw peninsula, Michigan, USA. The total flight call counts per site for springs only in spring 2022-spring 2024 listed next to each site; counts are also colored according to the relative size of the movement at each site.

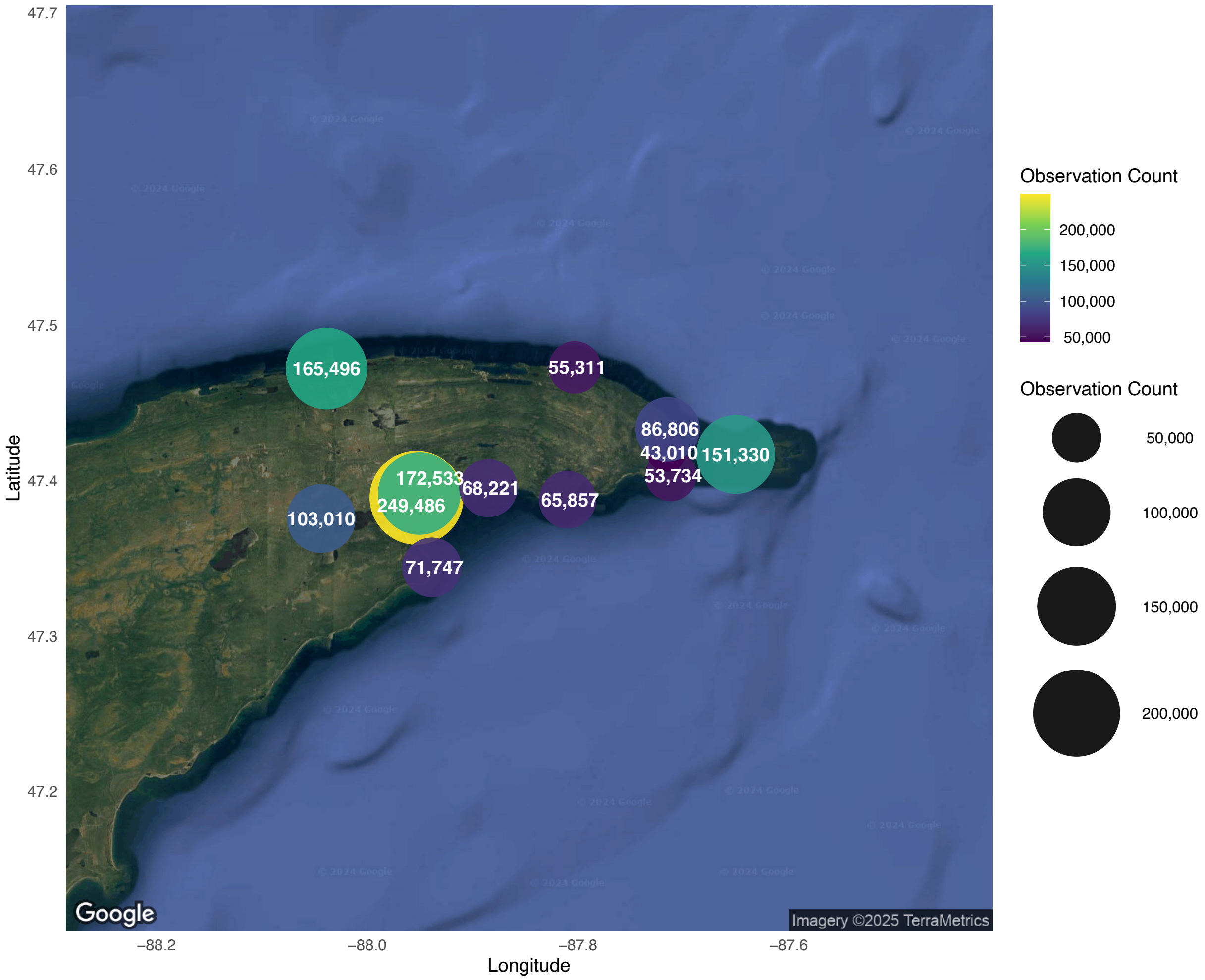

Supplemental Figure 12. Fall flight call detections of migratory Passeriformes engaged in diurnal reoriented migration events at 12 sites in the Keweenaw peninsula, Michigan, USA. The total flight call counts per site for falls only in fall 2022 and fall 2024 listed next to each site; counts are also colored according to the relative size of the movement at each site. For fall 2023, we are lacking data.

Supplemental Table 1. Total counts of Passeriformes, Parulidae, Passerellidae, and Turdidae at 12 acoustic recordings sites in the Keweenaw peninsula, Michigan, USA. Counts are summarized over five seasons between Spring 2022-Fall 2024.

|Site ID	Total detections	latitude	longitude
|Keweenaw Pt	111038	47.40353	-87.71181
|Manitou Island	379670	47.41699	-87.65025
|Bete Grise	514116	47.38918	-87.95305
|Agate Harbor	491155	47.47218	-88.03853
|Horseshoe Harbor	221578	47.47296	-87.80287
|Smith Fisheries	291644	47.39193	-87.95044
|Pt. Isabelle	160839	47.3444	-87.93858
|Bare Bluff	130246	47.39543	-87.88488
|Little Gratiot Ridge	244788	47.37583	-88.04359
|Gill Lake	109030	47.41837	-87.71573
|Rocket Range	203673	47.43326	-87.71483
|Big Bay	120697	47.38789	-87.80978
**Overall Total**	2978474		

Supplemental Table 2. Total counts of Passeriformes, Parulidae, Passerellidae, and Turdidae flight call detections at 12 acoustic recordings sites in the Keweenaw peninsula, Michigan, USA. Counts are summarized over five seasons between Spring 2022-Fall 2024.

|Taxonomic group	Total detections
**|Parulidae**	882,841
**|Passerellidae**	290,178
**|Passeriformes**	1,773,767
**|Turdidae**	31,688
**Total**	2,978,474

Supplemental Table 3. Model summary statistics for generalized additive mixed models of the effect of wind on Passeriformes Principal Components relating to Passeriformes landscape use intensity through the Keweenaw peninsula, Michigan. The slope coefficient for each model is presented along with asterisks indicating significance: 0 ***, 0.001 **, 0.01 *, 0.05., 00.1, 1. Included are also slope coefficients and significance for models on the effect of wind on Parulidae, Passerellidae, and Turdidae landscape use intensity through the Keweenaw peninsula, Michigan. Letters refer to A: mean east-west wind, B: mean north-south wind, C: AM change east-west wind, D: AM change north-south wind, E: nocturnal migration traffic, F: mean east-west wind: mean north-south wind, G: mean east-west wind: AM change north-south wind, H: mean east-west wind: nocturnal migration traffic, I: mean north-south wind: nocturnal migration traffic.

model	A	B	C	D	E	F	G	H	I
**Passeriformes Spring PC1**	-0.22*	0.0480	-0.13**	-0.0830	0.55***	0.0095	0.0028	0.0180	-0.0068
**Passeriformes Spring PC2**	-0.0230	-0.0380	0.0130	0.054*	0.0610	-0.0046	-0.0062*	0.0063	0.0110
**Passeriformes Spring PC3**	-0.0260	0.0051	0.0033	0.0430	0.11*	-0.0058*	-0.0016	0.0039	0.0034
**Passeriformes Fall PC1**	0.1800	0.1000	0.0034	0.0550	0.7***	-0.0150	0.0032	-0.0240	0.0044
**Passeriformes Fall PC2**	0.0280	0.0018	-0.0230	0.0088	0.0340	0.0047	0.0032	-0.0026	0.0000
**Passeriformes Fall PC3**	-0.0380	0.0410	0.0002	-0.0031	-0.0470	0.0004	0.0018	0.0025	-0.0058
**Parulidae Spring PC1**	0.1700	0.1300	0.14***	0.0480	-0.43***	-0.0045	-0.0016	-0.0130	-0.0240
**Parulidae Spring PC2**	-0.0032	-0.0590	0.0250	0.072**	0.15*	-0.0069*	-0.0063	0.0025	0.018*
**Parulidae Spring PC3**	-0.0430	-0.0160	-0.0098	0.057*	0.0940	-0.0032	0.0015	0.0055	0.0096
**Parulidae Fall PC1**	0.1400	0.0870	0.0092	0.0520	0.62***	-0.0066	0.0078	-0.0200	0.0081
**Parulidae Fall PC2**	-0.0300	0.0150	0.0240	-0.0210	-0.0710	-0.0038	-0.0023	0.0018	-0.0056
**Parulidae Fall PC3**	0.0012	0.0480	-0.0110	0.0060	-0.0640	0.0007	-0.0005	-0.0034	-0.0074
**Passerellidae Spring PC1**	-0.0730	-0.0300	-0.0650	-0.0650	0.33**	0.0054	0.0033	0.0063	0.0049
**Passerellidae Spring PC2**	-0.0450	-0.0240	0.0210	0.0340	-0.0760	0.0033	0.0018	0.013*	0.0074
**Passerellidae Spring PC3**	0.0100	0.0240	0.0038	0.0100	0.0740	-0.0067**	-0.0049	-0.0024	-0.0034
**Passerellidae Fall PC1**	-0.0130	0.1300	-0.0540	0.0130	0.4**	-0.0013	0.0052	0.0050	-0.0200
**Passerellidae Fall PC2**	0.0470	-0.0220	0.0029	0.0075	-0.0870	-0.0039	-0.0031	-0.0036	0.0007
**Passerellidae Fall PC3**	-0.0910	-0.0220	-0.0120	0.0017	-0.0850	-0.0013	-0.0035	0.0120	0.0042
**Turdidae Spring PC1**	-0.0180	-0.0260	0.0160	0.0410	-0.0650	0.0026	0.0063	0.0084	0.0028
**Turdidae Spring PC2**	-0.0061	-0.0003	-0.0034	-0.0120	0.0500	-0.0012	-0.0035	-0.0021	-0.0017
**Turdidae Spring PC3**	-0.0350	-0.0860	0.0330	-0.0240	0.0360	0.0054	0.0004	0.0079	0.0096
**Turdidae Fall PC1**	-0.1900	0.0910	0.0390	-0.0270	-0.51**	0.0089	0.0097	0.0310	-0.0240
**Turdidae Fall PC2**	-0.16*	-0.1200	-0.0620	-0.0680	0.0085	0.0038	0.0062	0.02*	0.0110
**Turdidae Fall PC3**	0.0170	-0.16*	-0.0080	-0.0430	-0.0500	-0.0054	0.0039	-0.0060	0.022**

Supplemental Table 4. AICc scores for top models examining effect of east-west component and north-south component wind on morning flight detections of Passeriformes in the Keweenaw peninsula, Michigan, USA in three different time bins throughout the night and morning.

	Time bin	AIC score (top model)
**Spring 2022**	midnight_to_5am_next_day	136612.3
	9pm_to_midnight_prior	136623.4
	6am_to_11am_next_day	136624.7
**Fall 2022**	midnight_to_5am_next_day	129235.2
	6am_to_11am_next_day	129248.4
	midnight_to_5am_next_day	129262.6
**Spring 2023**	midnight_to_5am_next_day	96013.33
	midnight_to_5am_next_day	96013.9
	midnight_to_5am_next_day	96014.19

Supplemental Table 5. Summary of generalized additive mixed model (GAMM) t-statistics for the effects of Lake Superior wind conditions and nocturnal migration traffic over Lake Superior on principal components representing landscape use by migrating Passeriformes in the Keweenaw peninsula, Michigan, USA. Principal components describe overall landscape use intensity (PC1), west–east gradients in landscape use (PC2), and north–south gradients in landscape use (PC3). Date of season was included as a smooth term; its significance is reported.

**Predictor variable**	**PC1 Spring**	**PC2 Spring**	**PC3 Spring**	**PC1 Fall**	**PC2 Fall**	**PC3 Fall**
**(Intercept)**	-4.78***	-1.48	-2.49*	-5.51***	0.48	1.28
**mean east-west wind**	-2.08*	-0.50	-0.60	1.48	0.54	-0.96
**mean north-south wind**	0.36	-0.66	0.10	0.68	0.97	0.88
**AM change east-west wind**	-2.79**	0.66	0.18	0.07	0.21	0.01
**AM change north-south wind**	-1.49	2.21*	1.89	1.04	0.65	-0.18
**nocturnal migration traffic**	4.85***	1.16	2.30*	5.69***	0.45	-1.25
**mean east-west wind:mean north-south wind**	1.49	-1.65	-2.23*	-1.79	0.13	0.15
**mean east-west wind:AM change north-south wind**	0.40	-2.07*	-0.56	0.37	0.33	0.62
**mean east-west wind:nocturnal migration traffic**	1.27	1.02	0.69	-1.51	0.65	0.48
**mean north-south wind:nocturnal migration traffic**	-0.39	1.52	0.48	0.23	1.00	-0.99
**date of season (smooth term)**		***	***	***		
**R^2**	0.34	0.36	0.18	0.46	0.11	0.002
